# *RPI-1* (human *DCDC2*) displays functional redundancy with Nephronophthisis 4 in regulating cilia biogenesis in *C. elegans*

**DOI:** 10.55730/1300-0152.2642

**Published:** 2022-11-21

**Authors:** Oktay İ. KAPLAN

**Affiliations:** Rare Disease Laboratory, School of Life and Natural Sciences, Abdullah Gül University, Kayseri, Turkey

**Keywords:** DCDC2, cilia, NPHP4, rare diseases

## Abstract

Projecting from most cell surfaces, cilia serve as important hubs for sensory and signaling processes and have been linked to a variety of human disorders, including Bardet-Biedl Syndrome (BBS), Meckel-Gruber Syndrome (MKS), Nephronophthisis (NPHP), and Joubert Syndrome, and these diseases are collectively known as a ciliopathy. DCDC2 is a ciliopathy protein that localizes to cilia; nevertheless, our understanding of the role of DCDC2 in cilia is still limited. We employed *C. elegans* to investigate the function of *C. elegans* RPI-1, a *Caenorhabditis elegans* ortholog of human DCDC2, in cilia and found that *C. elegans* RPI-1 localizes to the entire ciliary axoneme, but is not present in the transition zone and basal body. We generated a null mutant of *C. elegans rpi-1*, and our analysis with a range of fluorescence-based ciliary markers revealed that *DCDC2* and nephronophthisis 4 (NPHP-4/NPHP4) display functional redundant roles in regulating cilia length and cilia positions. Taken together, our analysis discovered a novel genetic interaction between two ciliopathy disease genes (RPI-1/DCDC2 and NPHP-4/NPHP4) in *C. elegans*.

## 1. Introduction

Cilia are cellular protrusions that extend outward from the majority of cells in the human body and are generally separated into three ciliary subcompartments: the basal body (BB), the transition zone (TZ), and the axoneme. The TZ at the base of the cilia serves as a barrier to govern the entry and exit of components into the cilia (Garcia-Gonzalo et al., 2011; Chih et al., 2012). The ciliary axoneme is a microtubule-based core that originates from the basal body and is surrounded by the ciliary membrane and is generally separated into two parts: proximal and distal, with the proximal part containing a microtubule doublet (both A and B tubules) and the distal part containing a microtubule singlet (only A tubule extensions). Cilia exist in two types: motile and nonmotile cilia, also known as primary cilium. Motile cilia use propelling forces to move cells or fluid flow (such as the motility of the unicellular green alga *Chlamydomonas reinhardtii*), whereas the primary cilium senses the extracellular environment (such as the sensation of urine flow by cilia in the kidney) and acts as a cellular hub for signaling pathways, including Hedgehog (Hh), Wnt, and platelet-derived growth factor receptor (PDGFRα) pathways (Anvarian et al., 2019). Cilia dysfunction or structural abnormalities cause cilia-related human conditions called ciliopathies, including Bardet-Biedl Syndrome (BBS), (Valente et al., 2014) Meckel-Gruber Syndrome (MKS) (Hartill et al., 2017), Nephronophthisis (NPHP) (Luo and Tao, 2018), and Joubert Syndrome (JBTS) (Parisi, 2009). Ciliopathies possess a wide range of phenotypic manifestations, including polydactyly, developmental delay, obesity, polycystic kidneys, and retinal degeneration. Proteomics, genetics analysis, and protein localization studies indicated the presence of several functional modules encompassing several disease genes at the TZ: The MKS module and the NPHP module (Sang et al., 2011; Williams et al., 2011; Yee et al., 2015; Li et al., 2016).

Cilia have a dedicated and unique bidirectional intraciliary transport system termed intraflagellar transport (IFT) for cilia assembly, maintenance, and function (Kozminski et al., 1993; Rosenbaum and Witman, 2002). IFT is made up of large protein complexes comprising kinesins, dynein, IFT-B, and IFT-A components. Kinesin-2 motors mediate the distribution of IFT cargos throughout cilia with the help of the IFT-B and IFT-A complexes, whereas ciliary cargos are removed from cilia by cytoplasmic dynein-2 motors that collaborate with the IFT-A complex (Blacque, 2008; Rosenbaum and Witman, 2002).

Human DCDC2 (doublecortin domain containing 2) causes a range of human diseases, including Nephronophthisis-related ciliopathies (NPHP-RC), human recessive deafness DFNB66, and Neonatal sclerosing cholangitis (NSC). (Grati et al., 2015; Schueler et al., 2015; Girard et al., 2016; Grammatikopoulos et al., 2016)*. DCDC2* was connected to dyslexia in 2005, however, a recent study indicated that deletion of DCDC2 did not enhance a risk factor for dyslexia (Meng et al., 2005; Scerri et al., 2017). DCDC2 was found to localize to cilia in a range of human and mouse cells but is excluded from the basal body (Grati et al., 2015; Schueler et al., 2015; Girard et al., 2016). DCDC2 was discovered to be associated with KIF3A, the IFT kinesin motor (Massinen et al., 2011). DCDC2 overexpression lengthens cilia and stimulates the Shh pathway, whereas DCDC2 downregulation has little effect on cilia length but impacts WNT signaling (Massinen et al., 2011). It is, however, uncertain why shRNA-mediated DCDC2 downregulation leads to no defect in cilia length, maybe because of partial downregulation.

Hence, we created a null mutant of RPI-1, the *Caenorhabditis elegans* ortholog of mammalian DCDC2, with CRISPR/Cas9 to investigate the exact role of DCDC2 in cilia (The Alliance of Genome Resources Consortium et al., 2020). In cilia, RPI-1/DCDC2 colocalizes with ciliary proteins such as CEPH-41 (the homolog of Joubert syndrome-associated CEP41) and IFT-140 (the homolog of human IFT140). While single *rpi-1* mutants exhibit no severe ciliary abnormalities, *rpi-1; nphp-4* (human nephrocystin-4) double mutants have a variety of ciliary structural defects, including short cilia, mispositioned cilia, and ectopic projections from the base of cilia. Our findings demonstrated the conserved role of *C. elegans RPI-1/DCDC2* in cilia biogenesis and first time revealed a novel genetic interaction between *RPI-1/DCDC2* and *NPHP-4*.

## 2. Materials and methods

### 2.1. *C. elegans* strains, maintenance, and genetic analysis

All worms were cultivated using the previously established standard techniques (Brenner, 1974). For mutant genotyping, polymerase chain reaction (PCR) was utilized. A null mutant for *rpi*-1, namely *rpi-1(syb722)* (*C. elegans* W07G1.5) was kindly generated by SunyBiotech with CRISPR/Cas9, and to exclude any background mutations, *rpi-1(syb722)* was outcrossed four times to the wild-type. *gpa-6prom::gfp, str-2prom::gfp*, *nphp-4(tm925) (*a component of NPHP module) and *bbs-5(gk537) (*a component of the BBSome) mutants were obtained from the *C. elegans* Genetic Center (CGC), while the Japanese National Bioresource Project provided the *mks-5(tm3100)* mutant (NBRP). *str-1prom::mCherry* was a kind gift from Piali Sengupta, Brandeis University, USA. Both MKS-6 and IFT-140 strains (vuaSi21[pBP39; Pmks-6::mks-6::mCherry; cb-unc119(+)]II.;mks-6(gk674) I and vuaSi24 [pBP43; Pche-11::che-11::mCherry; cb-unc-119(+)] II; unc-119(ed3) III; che-11(tm3433)V ) were gift from Peterman Lab and Dammermann Lab, respectively. To generate mutants with transgenic strains and double mutants, standard genetic crosses were performed.

Primers are listed below:

nphp-4(tm925) For: cagatttcgaagtgccagac; nphp-4 (tm925)
Rev: ctgagaacattcgataccag; mks-5 (tm3100)
For: ttcctcttgcagcatagccaag; mks-5 (tm3100)
Rev: tccacagtaaccatcctttgttcc; bbs-5 (gk537)
For: ttgcatgaatgtaccacttgcgg bbs-5 (gk537)
Rev : gaacctactcgcagggtgtc; rpi-1 (syb722)
For: ctagcacgatatgaatgactg, andrpi-1 (syb722)
Rev: ggtaatttcagcatctaagc.

### 2.2. Expression constructs and transgenic strains

Following the cloning of 500 bp of the *arl-13* promoter into the *C. elegans* expression vector pPD95.67 (modified vector), a 1317 bp cDNA *rpi-1* (W07G1.5) was inserted between the *arl-13* promoter and GFP, resulting in the *arl-13prom::rpi-1::gfp* expression construct. The *arl-13* promoter was chosen because it is exclusively expressed in ciliated sensory neurons in *C. elegans*. For microinjection, the 5ng/μL of *arl-13::rpi-1::gfp* was coinjected with 50 ng/μL RF4 (roller) and/or 1 ng/μL *ceph-41::prom::ceph-41::wrmpScarlet* (Cevik and Kaplan, 2021). Several transgenic lines expressing *arl-13::rpi-1::gfp* were generated with microinjection.

### 2.3. Confocal microscope analyses

The Zeiss LSM900 confocal microscope with Airyscan 2 coupled with ZEN 3 Blue edition software was utilized for colocalization analysis and other microscopy analyses. The high-resolution Z-stack images were acquired at 0.14 m intervals with a Plan ApoChromat 63x/1.40 NA, followed by Z-stack generation with Blue edition software ZEN 3, and the remainder were processed with ImageJ (NIH) software (Schindelin et al., 2012). A 3% agarose pad was created on microscope slides, followed by the application of 1 μL of 10 mM levamisole as an anesthetic agent before putting worms onto the agarose pads.

### 2.4. Osmotic avoidance assay

For the osmotic avoidance assay, a freshly prepared 8M fructose solution containing bromophenol blue was used to generate a ring (using 1 mL pipette tips) (Culotti and Russell, 1978). Both wild-type and cilia-deficient mutant *osm-5(p813)* worms were used as controls, and wild-type worms are anticipated to stay in the ring, but cilia-defective mutant *osm-5(p813)* worms are expected to cross the 8M bromophenol blue labeled ring. Each osmotic avoidance experiment, which takes 5 min, included five worms. Five worms were placed in the ring for each experiment, and the osmotic avoidance experiment was performed five times for each strain.

### 2.5. Dye-filling assay

A dye uptake assay was conducted as previously described (Perkins et al., 1986). Well-fed and healthy wild types and mutants were incubated in the lipophilic fluorescent dye for 45 min before being examined microscopically as previously reported (Kaplan et al., 2010).

### 2.6. Statistical analysis and plots

R was used to construct plots, and for the cilia length measurement, the Kruskal-Wallis test was used to evaluate whether or not there was a significant statistical distinction between wild type, single, and double mutants. The Chi-Square test was performed to calculate the statistical significance of osmotic avoidance between wild-type and mutants (*osm-5* and *rpi-1*). The codes used to generate statistical analyses have already been published (Turan, MG, et al., 2022), and the GitHub link is available here: https://github.com/thekaplanlab/Protocol-for-determining-the-average-speed-and-frequency-of-kinesin-and-dynein-driven-IFT.

## 3. Results

### 3.1. *C. elegans* DCDC2 localizes to the entire cilium in the sensory neurons

**Figure 1:**
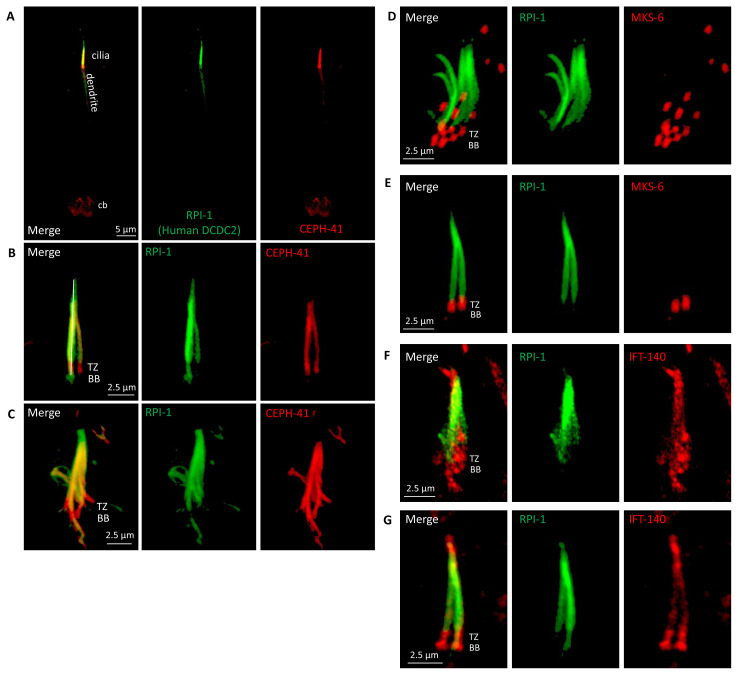
RPI-1/DCDC2 localizes to the entire axoneme in *C. elegans*. **A)** Shown is the colocalization of CEPH-41::wrmScarlet and RPI-1::GFP in *C. elegans*. RPI-1 is exclusively found in cilia. cb denotes the cell body. Dendrite is shown. **B and C)** RPI-1 localizes to the whole cilia of the ciliated sensory neurons in the amphid (head, lower panel) and phasmid (tail, upper panel) while CEPH-41 does not enter into the distal segment. **D, E, F, and G)** The localization of RPI-1 was further examined with MKS-6 (a transition zone marker) and IFT-140 (the ciliary IFT marker). No signal was observed in the transition zone most of the time. BB and TZ denote the basal body and transition zone respectively.

To determine the subcellular distribution of RPI-1 (a mammalian DCDC2 orthologue) in *C. elegans*, we tagged RPI-1 proteins with a green fluorescent protein (GFP) and coexpress fluorescent-tagged RPI-1 with ciliary axoneme and basal body marker IFT-140, transition zone marker MKS-6 or middle segment-specific marker CEPH-41 (Figure 1) (Mijalkovic et al., 2018; Cevik and Kaplan, 2021; Garbrecht et al., 2021). Transgenic strains coexpressing RPI-1::GFP and a red fluorescent-tagged ciliary marker were examined using a fluorescent confocal microscope to establish where RPI-1 is localized in *C. elegans* (Figure 1A). *C. elegans* RPI-1/DCDC2 appears to preferentially localize to cilia, with no enrichment in other cellular compartments, including cell body, dendrite, and axon, in the sensory neurons (Figure 1A**).** Close examination of RPI-1 reveals that RPI-1 localizes to the middle and distal segments (MS and DS) of amphid (head) and phasmid (tail) channels, and it does not colocalize with the transition zone marker MKS-6, suggesting that RPI-1 is not mostly detected in the transition zone (TZ) and basal body (BB) (Figures 1B, C, D, E, F, and G). Our cilia localization findings are consistent with previous reports that show DCDC2 localizes to cilia in human cells and *C. elegans* (Schueler et al., 2015; Grati et al., 2015; Jensen et al., 2016; Girard et al., 2016), but extend the initial findings by revealing that *C. elegans* RPI-1/DCDC2 is excluded from the TZ and BB. Taken together, these results suggest that RPI-1 localizes to the ciliary axoneme, but is absent from the TZ and BB.

### 3.2. Cilia length and morphology are unaffected in CRISPR-generated *DCDC2* loss of function mutant in *C. elegans*

**Figure 2:**
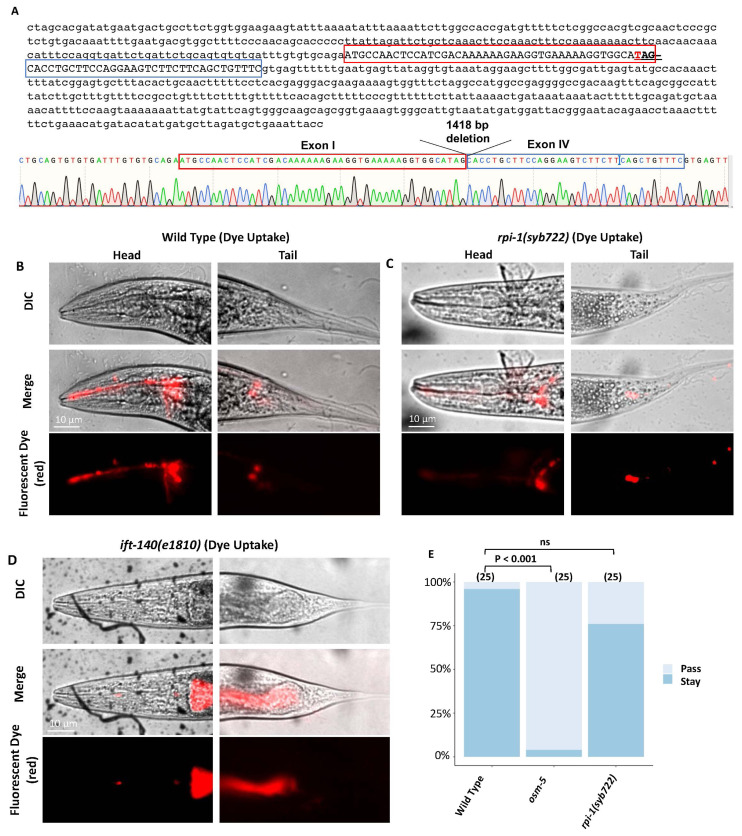

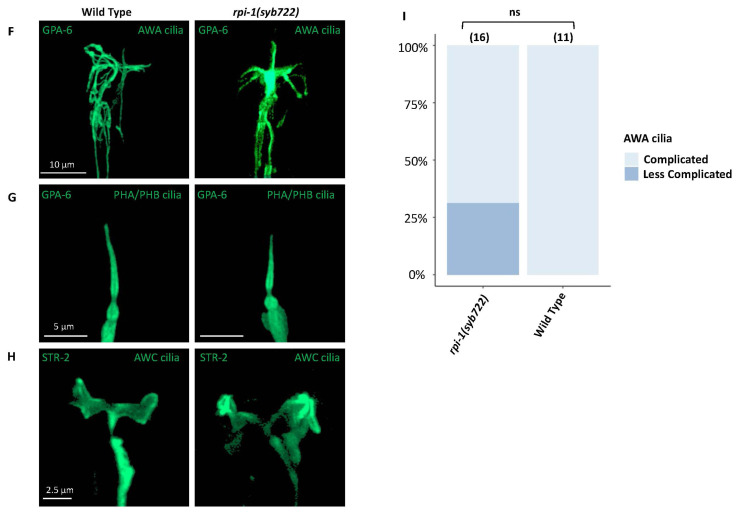
Structural analysis of cilia in *rpi-1* mutants. **A)** Shown are 1418 base pair deletion of *rpi-1* that removes the whole of Exon II and Exon III, leaving only 45 and 34 base pairs in Exon I and Exon IV, respectively. A single PCR result from the mutant strain was Sanger sequenced, and a representative electropherogram is shown. **B, C, and D)** The dye-filling assay was used to investigate the structure of the cilium. The capacity of *rpi-1* mutants to take up lipophilic fluorescent dye was assessed. The lipophilic fluorescent dye absorption was comparable to that of the wild type. More than 100 worms for each strain were counted. Shown are DIC, fluorescent, and merge images for wild-type, *ift-140(e1810)*, and *rpi-1* mutants. **E)** Functional analysis of cilia was further assessed with an osmotic avoidance assay. Unlike *osm-5* mutants, the majority of *rpi-1* mutants do not appear to pass osmotic barriers, suggesting that cilia function is likely unaffected. The Chi-Square test was used to examine the statistical significance of osmotic avoidance. The statistical comparisons between wild-type and mutants (*osm-5* and *rpi-1*) were presented in brackets, and NS denotes that the difference was not significant. The number indicates the number of animals utilized in the osmotic avoidance tests. **F, G, and H)** The structure of different types of the cilium was further examined with fluorescent-based markers for wild-type and *rpi-1* mutants. PHA/PHB is situated in the tail of *C. elegans* and extend a joint cilium. AWC and AWC cilia are found in the head of *C. elegans* and exhibit wing-like structures. The fluorescent images for AWA (*gpa-6prom::gfp*), AWC (*str-2prom::gfp*), and PHA/PHB (*gpa-6prom::gfp*) were depicted in wild-type and *rpi-1* mutants. There were no significant changes in PHA/PHB and AWC cilia structure between wild-type and *rpi-1* mutants, however, AWA cilia may be affected minimally in *rpi-1* mutants. **I)** Shown is a bar plot of AWA cilia analysis. Statistical analysis (the Chi-Square test) reveals that AWA cilia structure in *rpi-1* mutants was not significantly different from wild-type AWA cilia. The statistical significance between wild-type and *rpi-1* mutants is shown in brackets as “not significant” (NS). The number of AWA cilia analyzed was displayed.

Increases in ciliary length and activation of Shh signaling have been reported in over-expressed DCDC2 mammalian cells, but the shRNA-mediated reduction of DCDC2 did not affect ciliary length (Massinen et al., 2011). However, the lack of effect on cilia length might be due to incomplete *DCDC2* downregulation by shRNA. To determine whether the unexplored *C. elegans* ortholog of DCDC2 has a function in cilia formation in *C. elegans*, we created a null RPI-1/DCDC2 mutant with CRISPR/Cas9 in *C. elegans*. *C. elegans* RPI-1/DCDC2 has four exons and our *RPI-1/DCDC2* mutant (namely *rpi-1(syb722)*) possesses 1418 base pair deletion, removing entire exon II and III, as well as the majority of exon I and IV and it is a likely a null allele of *RPI-1* (Figure 2A).

The next step is to conduct a dye-filling experiment to test if *rpi-1* mutants display any structural defects in cilia. The dye-filling assay has been used to assess the presence of structural defects in cilia (Perkins et al., 1986; Starich et al., 1995). In summary, wild-type worms dye their ciliated sensory neurons by absorbing the lipophilic fluorescent dye through their cilia, but mutants with abnormal cilia structures, such as *ift-140(e1810)* (Intraflagellar transport 140), are unable to stain their ciliated sensory neurons due to structural defects in cilia. We found that similar to wild-type, *rpi-1* mutants soak up the lipophilic fluorescent dye into cells, suggesting there are no gross structural defects in cilia (Figures 2B, C, and D). In *C. elegans*, there are different types of cilia in the head and tail. AWC and AWC sensory neurons in the head have wing-like cilia at the dendrite end, whereas PHA/PHB sensory neurons in the tail have a joint extended cilium. We then picked AWA (marked with *gpa-6promoter::gfp*), PHA/PHB (marked with *gpa-6promoter::gfp*), and AWC (marked with *str-2promoter::gfp*) to examine individual cilia structure and morphology. These fluorescence-based markers illuminate specific cilia structures and we crossed them into *rpi-1(syb722)* mutants. Confocal microscopy analysis revealed that cilia morphology was generally unaffected in *rpi-1* mutants, but we observed some structural abnormality in AWA cilia (Figure 2F, G and H, I**).** Specifically, wild-type AWA cilia project multiple branches, whereas some *rpi-1* mutants have fewer branches in AWA cilia (Figure 2F and I). Our fluorescence-based marker analysis was consistent with normal dye uptake. In addition, our osmotic avoidance experiment, which measures cilia function, demonstrated that *rpi-1(syb722)* mutants possess functional cilia (Figure 2E).

### 3.3. DCDC2 and nephronophthisis 4 regulate cilia morphology in a functionally redundant manner in *C. elegans*

**Figure 3:**
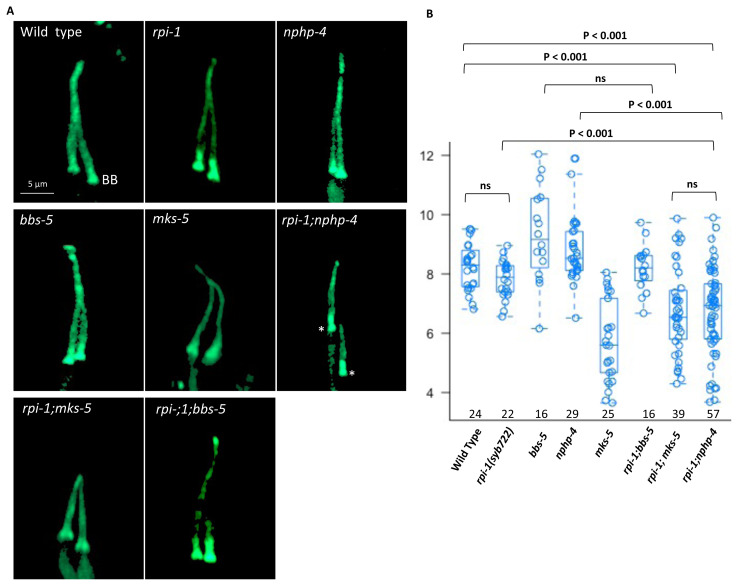
Phasmid cilia are short in *rpi-1; nphp-4* double mutants. **A)** Cilia structure analysis of double and single mutants was made by imaging phasmid cilia (PHA/PHB cilia). Asterisk points to the basal body and cilia are mispositioned in *rpi-1; nphp-4* double mutants. PHA/PHB cilia in the mutants were imaged using the fluorescent marker IFT-74::GFP. **B)** Cilia length was measured in the wild-type and single and double mutants. Cilia in *rpi-1; nphp-4* double mutants are shorter than in wild-type and single mutants. The numbers represent the number of cilia measured. To determine statistical significance, the Kruskal-Wallis test was utilized, and the brackets indicate statistical comparisons between mutants and/or wild-types. NS stands for not significant.

Because oligogenic inheritance has been reported for ciliopathies, and mutations in human *DCDC2* have been linked to nephronophthisis-related ciliopathies (NPHP-RC), we generated double mutants of *rpi-1* with ciliopathy gene ortholog mutants (Nephronophthisis, Bardet Biedl syndrome and Meckel–Gruber syndrome) to investigate the genetic interactions (Hoefele et al., 2007; Schueler et al., 2015). We picked the mutants *bbs-5(gk537)* (a component of BBSome), *mks-5(tm3100)* (a component of MKS module), and *nphp-4(tm925)* (a component of NPHP module) because they are all ciliopathy disease genes, they do not have severe defects in cilia structure, and structural enhancement in cilia would be visible if they do. BBS-5 is a BBSome component that is found in the ciliary axoneme, whereas MSK-5 and NPPH-4 are transition zone proteins. We chose PHA/PHB cilia for genetic analysis and generated single and double mutants expressing IFT-74::GFP (an endogenously tagged fluorescence IFT protein) (Yi et al., 2017). Confocal microscopy analysis of fluorescence-based marker revealed that double mutants of *rpi-1* with *bbs-5(gk537)* or *mks-5(tm3100)* showed no additive defects in cilia length and cilia morphology, however, *rpi-1; nphp-4(tm925) C. elegans* double mutant exhibited several structural abnormalities in cilia (short and mispositioned cilia, ectopic project) (Winkelbauer et al., 2005; Jauregui and Barr, 2005; Yee et al., 2015; Li et al., 2016). We found that the PHA/PHB cilia length of *rpi-1; nphp-4* double mutant is 13% shorter than that of the wild type (wild type = 8.24 μm and *rpi-1; nphp-4* = 7.22 μm) (Figure 3A and B). Furthermore, we noticed that the *rpi-1; nphp-4* doubled mutant has mispositioned PHA/PHB cilia (Figure 3A).

**Figure 4:**
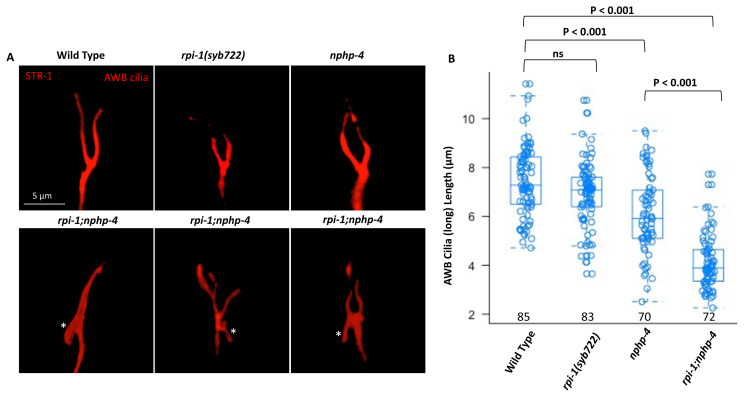
Ectopic projection from the ciliary base in *rpi-1; nphp-4* double mutants **A)** The fluorescent marker *str-1prom::mCherry* was used to examine AWB cilia structure in wild type, *rpi-1, nphp-4 s*ingle, and *rpi-1; nphp-4* double mutants. The structure of AWB cilia was altered in *rpi-1; nphp-4* double mutants with ectopic projection from the base of AWB cilia. **B)** The length of the AWB cilia (long cilia) was measured in the designated mutants, and the AWB cilia in *rpi-1; nphp-4* double mutants is shorter than in the wild type and single mutants. Numbers indicate the number of AWB cilia lengths measured. Statistical significance was determined with the Kruskal-Wallis test and the brackets showed which mutants and/or wild types were statistically compared. NS denotes not significant.

We sought to expand on the initial findings in PHA/PHB cilia, thus we created a *rpi-1; nphp-4* double mutant expressing the AWB-specific fluorescence-based marker (*str-1promoter::mCherry*) to examine different cilia in this double mutant. Similar to the findings of PHA/PHB, our examination showed that the AWB cilia (long cilia) of the *rpi-1; nphp-4* double mutant are shorter than the wild type or either single mutant (Figure 4A and B). Furthermore, we noticed that the *rpi-1; nphp-4* double mutant has an ectopic projection from the base of AWB cilia. Taken together, our findings indicate that *nphp-4* and *rpi-1* act in parallel pathways to regulate cilia length and cilia positioning.

## 4. Discussion

DCDC2 is a Doublecortin Domain Containing 2 known to cause human diseases, including Nephronophthisis-related ciliopathies (NPHP-RC), human recessive deafness DFNB66, and Neonatal sclerosing cholangitis (NSC) (Grati et al., 2015; Schueler et al., 2015; Girard et al., 2016; Grammatikopoulos et al., 2016). DCDC2 has been reported to localize to cilia, and DCDC2 overexpression impacts cilia length in humans (Massinen et al., 2011; Grati et al., 2015; Schueler et al., 2015; Girard et al., 2016). Both the human and C. elegans DCDC2 protein belong to a doublecortin domain-containing family, and the doublecortin domain has been linked to microtubule polymerization (Horesh et al., 1999). Here, we characterize a null mutant of *rpi-1*, a *C. elegans* ortholog of human DCDC2, in *C. elegans*. Genetic analysis is a powerful approach to revealing genetic interactions between two genes, and our genetic analysis helps us to uncover a novel genetic interaction between two ciliopathy disease genes (RPI-1/DCDC2 and NPHP-4/NPHP4) in *C. elegans*. This novel genetic interaction was revealed by analyzing cilia length and morphology in single and *rpi-1; nphp-4* double mutants.

Normally, NPHP-4 and RPI-1 are single mutations that do not play essential roles in determining cilia length and positioning. Combination mutations, on the other hand, cause severe ciliary abnormalities, including cilia length and cilia positioning, highlighting the importance of these components for cilia length and cilia position. Such genetic interactions revealed previously undiscovered *RPI-1* functions.

Similar ciliary abnormalities (short cilia and cilia positioning defects) were previously reported in mutants lacking two TZ components (*mks-6* and *nphp-4*) from the MKS and NPHP modules (Williams et al., 2011). However, the genetic connection of *DCDC2* with *NPHP-4*, a transition zone protein, is surprising because NPHP-4 is a component of the Y-links and the DCDC2 protein is found in the ciliary axoneme in both humans and *C. elegans*, excluded from the transition zone. How these two distinct localizing proteins interact with one another remains to be determined.

On the other hand, Reiter and colleagues previously reported genetic interactions between *bbs-5(gk507) (*a component of the BBSome and axoneme localizing protein) and *nphp-4(tm925)* in regulating cilia morphology, implying that *nphp-4* may act in a parallel pathway with two different axonemal proteins (RPI-1 or BBS-5) to regulate cilia morphology (Yee et al., 2015). While *bbs-5* and *rpi-1* interact genetically with *nphp-4*, we demonstrated that simultaneous deletion of *rpi-1* and *bbs-5* did not disrupt ciliary structure in *C. elegans*.

Mutations in human *DCDC2* were implicated in Nephronophthisis-related ciliopathies (NPHP-RC), human recessive deafness DFNB66, and Neonatal sclerosing cholangitis (NSC). The implications of our findings for these disorders are somewhat unclear, but the oligogenic inheritance was reported for human ciliopathies, including Bardet–Biedl syndrome (BBS). Furthermore, the clinical characteristics were influenced by a variety of genetic modifiers (Perea-Romero et al., 2022). We believe our findings may offer some kind of clue regarding oligogenic gene combinations and modifiers. For example, could *NPHP4* and *DCDC2* genes act as genetic modifiers for one another in humans? We can speculate mutations in these two genes might lead to oligogenic inheritance in human ciliopathies. Consistent with this speculation, Yoder and colleagues demonstrated that genetic interactions between *bbs-5* and *nphp-4* in *C. elegans* are conserved in mammals (Bentley-Ford et al., 2022). Importantly, the conserved genetic interactions between *bbs-5* and *nphp-4* in mammals suggest that the observed genetic interactions between *rpi-1* and *nphp-4* may also be preserved in mammals. To fully comprehend the significance of these genetic connections, more research is required.
